# Genotyping and investigating capsular polysaccharide synthesis gene loci of non-serotypeable *Streptococcus suis* isolated from diseased pigs in Canada

**DOI:** 10.1186/s13567-017-0417-6

**Published:** 2017-02-20

**Authors:** Han Zheng, Xiaotong Qiu, David Roy, Mariela Segura, Pengchen Du, Jianguo Xu, Marcelo Gottschalk

**Affiliations:** 1 State Key Laboratory for Infectious Disease Prevention and Control, Collaborative Innovation Center for Diagnosis and Treatment of Infectious Diseases, National Institute for Communicable Disease Control and Prevention, Chinese Center for Disease Control and Prevention, Changping, Beijing, China; 2 Faculty of Veterinary Medicine, Swine and Poultry Infectious Diseases Research Center, University of Montreal, Quebec, Canada; 3 Institute of Infectious Diseases, Beijing Ditan Hospital, Capital Medical University, Beijing Key Laboratory of Emerging Infectious Diseases, Beijing, People’s Republic of China

## Abstract

*Streptococcus suis* (*S. suis*) is an important swine pathogen and an emerging zoonotic agent. Most clinical *S. suis* strains express capsular polysaccharides (CPS), which can be typed by antisera using the coagglutination test. In this study, 79 *S. suis* strains recovered from diseased pigs in Canada and which could not be typed using antisera were further characterized by capsular gene typing and sequencing. Four patterns of *cps* locus were observed: (1) fifteen strains were grouped into previously reported serotypes but presented several mutations in their *cps* loci, when compared to available data from reference strains; (2) seven strains presented a complete deletion of the *cps* locus, which would result in an inability to synthesize capsule; (3) forty-seven strains were classified in recently described novel *cps* loci (NCLs); and (4) ten strains carried novel NCLs not previously described. Different virulence gene profiles (based on the presence of *mrp*, *epf*, and/or *sly*) were observed in these non-serotypeable strains. This study provides further insight in understanding the genetic characteristics of *cps* loci in non-serotypeable *S. suis* strains recovered from diseased animals. When using a combination of the previously described 35 serotypes and the complete NCL system, the number of untypeable strains recovered from diseased animals in Canada would be significantly reduced.

**Electronic supplementary material:**

The online version of this article (doi:10.1186/s13567-017-0417-6) contains supplementary material, which is available to authorized users.

## Introduction

*Streptococcus suis* is recognized as one of the most important causes of bacterial disease in post-weaned piglets worldwide, generating important economic losses to the swine industry. In addition, it is an important emerging zoonotic agent [1–3]. Clinical strains of *S. suis* generally have a capsule (capsular polysaccharide or CPS), which is the basis of the serotyping traditionally used for epidemiological studies. Thirty-five serotypes of *S. suis* (serotype 1 through 34 and serotype 1/2) were identified in the 1980s and the 1990s [4–7]. More recently, serotypes 20, 22, 26, 32, 33 and 34 have been suggested as belonging to a species different from *S. suis* [8, 9]. Strains isolated from diseased pigs primarily belong to serotype 2 in most countries, followed by serotypes 3, 4, 5, 7, 8 and 1/2 [10–12]. In some European countries, serotype 9 is also one of the most frequently recovered capsular types from diseased animals [12, 13]. Traditionally, *S. suis* is routinely serotyped by the coagglutination test using serotype-specific antisera. However, non-serotypeable *S. suis* strains are frequently reported in many studies [12, 14–18]. Given that strains not expressing the CPS cannot be serotyped using antisera, serotyping based on molecular techniques has been proposed. Since the *S. suis* CPS is synthesized by the Wzx/Wzy pathway in the CPS locus, *wzy* genes have been demonstrated to be serotype-specific [19]. Thus, high-throughput capsular gene typing systems based on serotype-specific *wzy* genes have become attractive alternatives/complement to the existing serological tests [18, 20, 21]. However, even with the use of multiplex PCR tests, non-serotypeable strains are still commonly isolated from both clinically healthy and diseased animals [18, 22, 23].

In recent years, 17 novel *cps* loci (NCLs) were identified from non-serotypeable *S. suis* and were designated as NCL1 to 16 and serotype Chz [22–24]. Meanwhile, an 18-plex Luminex assay was also developed to detect these 17 NCLs and nearly 60% of non-serotypeable strains from healthy pigs carried one of these NCLs [22]. However, little is known about the distribution and characteristics of the *cps* loci of potentially virulent non-serotypeable strains recovered from diseased animals.

In this study, the *cps* loci of 79 Canadian non-serotypeable *S. suis* strains (as determined by the coagglutination test) recovered from diseased pigs were studied using two capsular gene typing systems [20, 22] and the genetic characteristics of the NCLs were analyzed. To elucidate the non-serotypeable mechanisms of strains grouped into previously described serotypes, the study was extended to compare their *cps* sequence to that of corresponding reference strains. Furthermore, the prevalence of minimum core genome (MCG) sequence typing group and virulence gene profile were also investigated in all 79 strains.

## Materials and methods

### Bacterial strains and chromosomal DNA preparation

A total of 79 *S. suis* strains isolated from diseased pigs on non-related farms in Canada were used in this study (Additional file 1). All strains have been isolated from primary affected organs of clinically diseased pigs, including brain (meningitis; *n* = 18), heart (endocarditis; *n* = 18), multiple organs (septicemia; *n* = 14), pleura (polyserositis; *n* = 9), lungs (pneumonia; *n* = 9) and joints (arthritis; *n* = 1). For a very few isolates, the information was not available, but they were all recovered from diseased animals with a primary diagnosis of *S. suis* infection. All isolates were serotyped using the coagglutination test [25]. Chromosomal DNA was prepared from all strains as previously described [21]. The species identity of the 79 strains was determined to be *S. suis* by amplification of the 16S rRNA, *recN*, *gdh*, and *thrA* genes [20, 26–28].

### Capsular gene typing

The *cps* locus type of the 79 strains was identified by the 32-plex and 18-plex Luminex assays previously reported [20, 22]. The subtypes of known NCLs were determined based on the arrangement of subtype-specific homology groups (HGs) and transposases [22, 23].

### Sequencing *cps* loci and bioinformatics analyses

Seventeen strains which could not be grouped using the 32- and 18-plex Luminex assays and 3 strains which could not be grouped into known subtypes, as well as 15 strains which were grouped into reference serotypes, were sequenced by Illumina sequencing as previously described [23]. Each *cps* locus sequence was extracted from the draft genome sequence and was analyzed using the same bioinformatics methods described in previous studies [19, 22, 23]. The products of the *cps* genes were assigned to novel HGs if both of the global match regions and identity of the amino acid or nucleotide sequences were below 50% when compared to the 420 currently known HGs of the 35 reference serotypes and 17 NCLs. The novel HGs were assigned numerical values from HG421 onwards [19, 22, 23]. Novel HGs that were present in all strains of a given NCL were identified as NCL-specific HGs. The strains harboring the same *wzy* were clustered into the same NCL. The Artemis comparison tool (ACT) was used to visualize the data [29].

### MCG typing and PCR assays for *mrp* (muramidase released protein), *sly* (suilysin) and *epf* (extracellular protein)

MCG sequence typing was performed using PCR amplification and DNA sequencing as previously described [30, 31]. The full-length *mrp* gene was amplified and sequenced using a previously described method [11]. Amplification of the *sly* and *epf* genes was performed according to methods previously described [32, 33].

### Nucleotide sequence accession number

Sequences of *cps* loci obtained in this study were deposited in GenBank under the accession numbers KX870047–KX870056, KX870058–KX870064, KX870067–KX870072, and KX870074–KX870076. Reads of the sequenced strains were deposited in GenBank under accession number SRR5177663–SRR5177696 and SRR5177711. All accession numbers can also be found in Additional file 1.

## Results

### Serotyping of strains

The 79 strains used in the present study showed auto-agglutination, poly-agglutination or non-agglutination using the reference antisera and the coagglutination test and were thus considered as non-serotypeable. All strains were then typed using our previously developed capsular gene typing systems [20, 22]. Fifteen strains (18.9%) were grouped into reference serotypes while 47 (59.4%) were grouped into 17 known NCLs. The remaining 17 strains remained non-typeable (Additional file 1).

Of the 15 strains belonging to the previously described serotypes, serotype 2 or 1/2 (*n* = 4), which cannot be distinguished by capsular gene typing, was the most frequent, followed by serotypes 15 (*n* = 3), 11 (*n* = 2), and 30 (*n* = 2). Serotypes 5, 17, 27 and 29 only contained a single strain (Additional file 1).

Of the 47 strains which were assigned to previously known NCLs, NCL3 (*n* = 18) was the most prevalent, followed by NCL4 (*n* = 8), NCL7 (*n* = 4), NCL2 (*n* = 3), NCL12 (*n* = 3), and NCL13 (*n* = 3). In addition, one strain each of the NCL1, 5, 6, 10, 11, 14, 16, and Chz were also found (Additional file 1).

### Identification of four new NCLs

The remaining 17 non-serotypeable strains mentioned above were sequenced by Illumina sequencing. The *cps* locus was absent from 7 strains. The *cps* loci of the remaining 10 strains were divided into four new NCLs which were named NCL17 to 20 based on their *wzy* gene sequences. NCL17 was the most prevalent (*n* = 4), followed by NCL18 (*n* = 3), NCL19 (*n* = 2), and NCL20 (*n* = 1) (Additional file 1). In addition, two types of patterns were found in the four new NCLs. NCL17 and NCL18 belonged to pattern I-a, while NCL19 and NCL20 belonged to pattern I-b [19].

**Figure 1:**
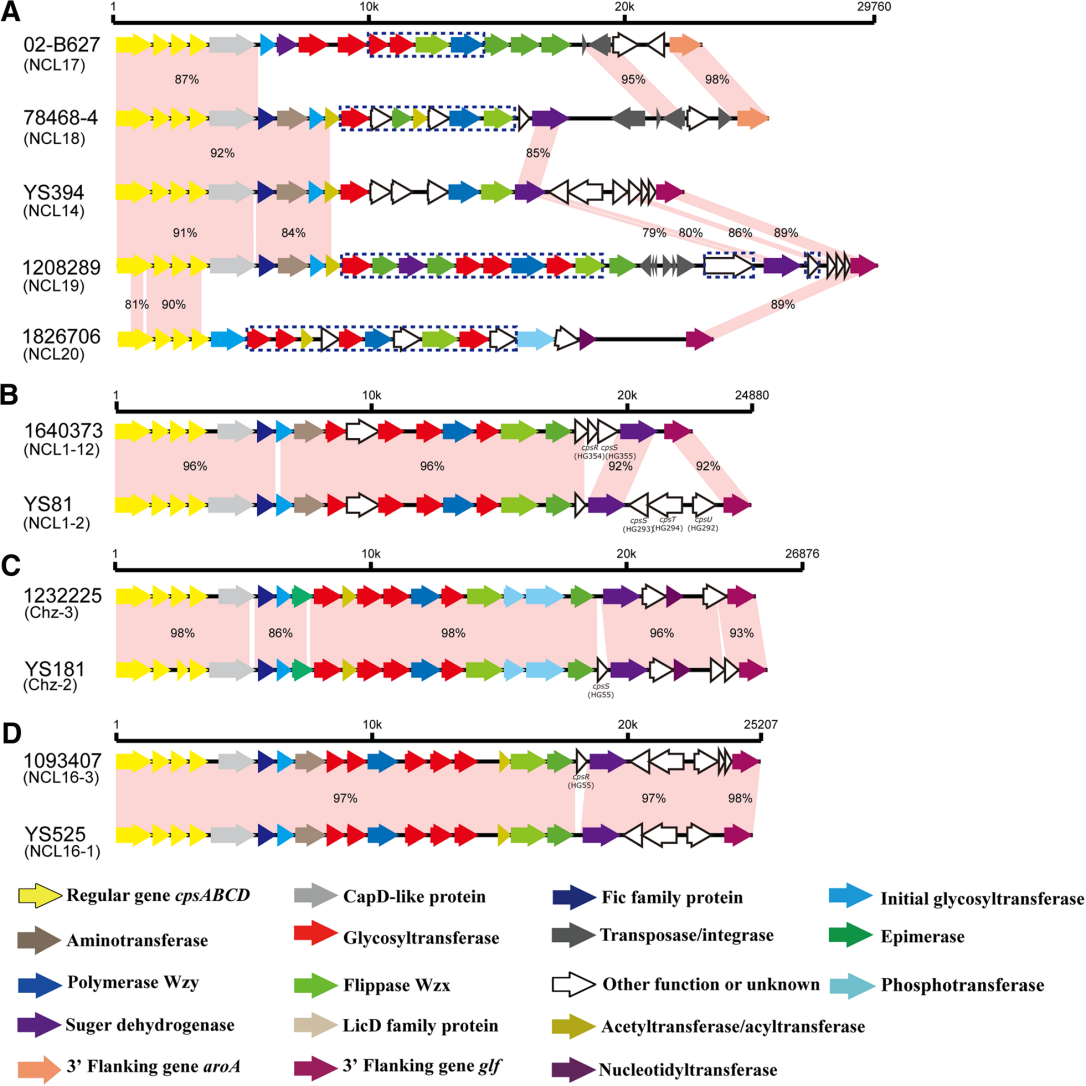
**Comparison of the** ***cps*** **loci among NCL17 to 20 (A) and within NCL1 (B), Chz (C) and NCL16 (D).** Each colored arrow represents the gene whose predicted function is shown in the below panel. NCL-specific genes are indicated by dotted blue lines.

The sizes of these NCLs ranged from 21.34 to 29.90 kb and the percentage of G+C content varied between 33.9 and 35.1%. Fifty-nine predicted coding sequences were designated *cps* HGs. Twenty-two HGs were also present in the *cps* loci of the reference strains of known serotypes and 17 known NCLs. An initial sugar transferase gene was located in the 5′ region and was classified into three HGs: HG6 (NCL20), HG8 (NCL17), and HG295 (NCL18 and NCL19). The 5′ regions of four NCLs were conserved, whereas the central and 3′ regions of these were highly variable (Figure 1A).

Thirty-two HGs were NCL-specific. Each NCL contained 4–11 NCL-specific genes, with 4 HGs for NCL17, 7 HGs for NCL18, 11 HGs for NCL19, and 10 HGs for NCL20. Among these, 11 HGs encoded putative glycosyl transferases and two encoded acetyltransferases. As expected, all Wzy polymerases and Wzx flippases were NCL-specific (Additional file 2).

### Determining the subtypes of NCLs

NCL2, NCL3, NCL7, and NCL11 strains were found to belong to a single subtype; NCL2-4, NCL3-1, NCL7-1, and NCL11-5, respectively. Genetic heterogeneity was not found within strains of NCL12, NCL15, and NCL17 to 20 (Additional file 2).

i. NCL1: strain 1640373 could not be classified into any known NCL1 subtype and was sequenced by Illumina sequencing, named as NCL1-12. The replacement of HG293, HG294, and HG292 by the HG354 and HG355 was found in its three side regions (Figure 1B).
ii. Chz: compared to the reference strain Chz-2, the deletion of HG55 was found in strain 1232225, named as Chz-3 (Figure 1C).
iii. NCL16: compared to the reference strain YS525 (NCL16-1), the insertion of HG55 was found in strain 1093407, named NCL16-3 (Figure 1D).

### Mutations in the *cps* loci of strains belonging to previously described serotypes

**Figure 2:**
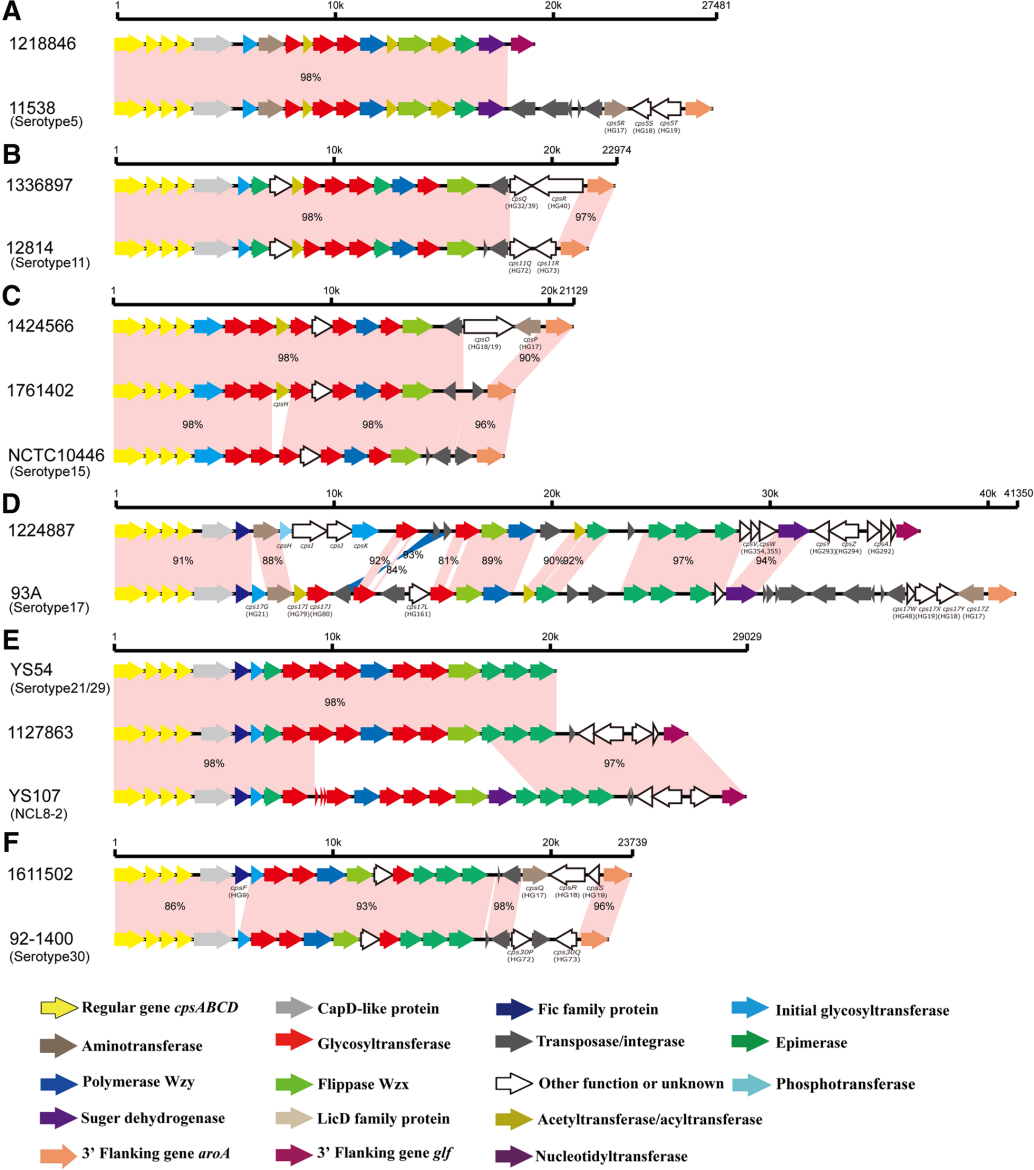
**Comparison of the** ***cps*** **loci within serotype 5 (A), serotype 11 (B), serotype 15 (C), serotype 17 (D), serotype 21/29 (E) and Chz (F).** Each colored arrow represents the gene whose predicted function is shown in the below panel.
iii. Serotype 11: compared to the serotype 11 reference strain 12814 (GenBank accession number AB737819), HG72 and HG73 were replaced by HG32 and HG40 in strains 1336897 and 1336915. In addition, the nucleotide substitutions (TA→CC) of the termination codon of HG32 were found in the *cpsQ* gene of strains 1336897 and 1336915, which resulted in the chimeric HG32/HG39 gene (Figure 2B).
iv. Serotype 15: two types of variations were found within this serotype. Strains 1424566 and 1449343 possessed identical *cps* sequences. A novel HG (*cps*H, putative acetyltransferase) was inserted between HG33 and HG77, and the insertions of HG19 and HG17 at the 3′ end were found in two strains (Table 1). Moreover, the transversion (T→G) was found at the site of the termination codon of HG19, which resulted in the chimeric HG18/HG19 gene in two strains. Compared to strains 1424566 and 1449343, HG18 and HG17 were replaced by a transposase in strain 1761402 (Figure 2C).

The 15 strains that were negative by coagglutination test but positive by multiplex Luminex assay for the reference serotypes were further analyzed. Comparing to the *cps* locus of the corresponding serotype reference strains, insertions and deletions were found in the serotype 5, 11, 15, 17 and 30 strains. The *cps* loci of four serotype 2 or 1/2 strains and one serotype 27 strain were intact and small-scale mutations were detected in these (Table 2).

i. Serotype 2 or 1/2: compared to the serotype 2 reference strain P1/7 (GenBank accession number BR001000), all four strains had a 33 bp insertion in *wxy* genes and four strains had single-nucleotide substitutions in *wzx* genes. The single-nucleotide substitutions in glycosyltransferase genes and a 27 bp deletion in the side-chain formation gene were also found in five strains (Table 2).
ii. Serotype 5: compared to the serotype 5 reference strain 11538 (GenBank accession number BR001003), the deletions of HG17 to HG19 at the 3′ end were found in strain 1218846 (Figure 2A).

**Table 1:** **Information of the novel HGs inserted** ***cps*** **loci of strains belonging to reference serotypes**

Strain ID	*cps* locus type	Gene name	Predicted products	Similar protein, species (GenBank accession number)	Coverage/identity (%)
1424566, 1449343, 1761402	Serotype 15	*cpsH*	Acetyltransferase	Maltose O-acetyltransferase, *Lactobacillus reuteri* (CUR43586.1)	96/68
1224887	Serotype 17	*cpsH*	UDP-phosphate galactose phosphotransferase	UDP-phosphate galactose phosphotransferase, *Sphaerochaeta pleomorpha* (WP_014270310.1)	99/57
		*cpsI*	Hypothetical protein	Biotin carboxylase, *Sphaerochaeta pleomorpha* (WP_014270309.1)	68/48
		*cpsJ*	Biotin carboxylase	Biotin carboxylase, *Ruminococcus* sp. (CBL19829.1)	99/60
		*cpsK*	Glycosyltransferase	Glycosyltransferase family 1 protein, *Gallibacterium anatis* (WP_065231950.1)	92/58
v. Serotype 17: compared to the serotype 17 reference strain 93A (GenBank accession number AB737824), two deletions (HG21 and HG161) and two insertions (HG354 and HG355) were found. Furthermore, HG79 and HG80 were replaced by a putative phosphotransferase, a putative hypothetical protein, a putative biotin carboxylase, and a putative glycosyltransferase (initial sugar transferase), which were not assigned to any previous homology group (Table 1). Moreover, the replacement of HG48, HG17, HG18, and HG19 by HG293, HG294, and HG292 was also found (Figure 2D).
vi. Serotype 27: compared to the serotype 27 reference strain 89–5259 (GenBank accession number AB737831), the single-nucleotide substitutions and small-scale indels in glycosyltransferase genes, *wzx* gene, and side-chain formation gene were found (Table 2).

**Table 2:** **Mutations in glycosyltransferase, side-chain formation,** ***wzy*** **and** ***wzx*** **genes of serotype 2 or 1/2 representative strains and a serotype 27 strain**

Strain	Affected gene(s)	Types of mutations	Affected nucleotide(s) [Affected amino acid]
1827702	*cps2E*	Missense	A61G [Thr21Ala]
	*cps2I*	Insertion	IS element: 33 bp
	*cps2N*	Deletion	27 bp
1090772	*cps2E*	Missense	A61G [Thr21Ala]
	*cps2F*	Missense	A149G [Asp50Gly]
		Missense	A1047T [Leu349Phe]
	*cps2I*	Insertion	IS element: 33 bp
	*cps2N*	Deletion	27 bp
	*cps2O*	Missense	C859A [Arg287Ser]
1160406	*cps27E*	Missense	A506G [Asp169Gly]
		Missense	T508A [Ser170Thr]
		Missense	A513T [Glu171Asp]
		Missense	A522T [Lys174Asn]
		Missense	A524T A525T [Lys175Ile]
		Missense	A541C C543G [Ile181Leu]
		Missense	A553G [Ile185Val]
		Missense	G617T T618G [Ser206Met]
		Missense	A623T [Tyr208Phe]
		Missense	T633G [Leu211Val]
		Missense	C640T A642C [Leu214Phe]
		Missense	A651T [Glu217Asp]
		Missense	C692T [Ser231Leu]
		Missense	G706A A708G [Ala236Thr]
		Missense	G874A A876T [Val292Ile]
		Missense	T905C [Val302Ala]
		Missense	A922C A924G [Lys308Gln]
		Missense	A941C G942A [Lys314Thr]
		Missense	A967G [Ile323Val]
		Missense	A997C G999C [Met333Leu]
		Missense	G1000A C1001G T1002C [Ala334Ser]
		Missense	A1174C A1175G [Lys392Arg]
		Missense	G1186A G1187T T1188G [Gly396Met]
		Missense	A1272C [Glu424Asp]
		Missense	A1279C A1280G [Lys427Arg]
		Missense	G1288T T1290A [Val430Leu]
		Missense	A1292C [Glu431Ala]
		Missense	G1324A A1326T [Val442Ile]
		Missense	A1357T T1359G [Ile453Leu]
		Missense	A1360T A1361T [Lys454Leu]
	*cps27F*	Deletion	27 bp
		Missense	A662T [His221Leu]
	*cps27G*	Insertion	IS element: 21 bp
	*cps27I*	Missense	G483C [Trp161Cys]
		Missense	C513A [Asp171Glu]
		Missense	A611G [Gln204Arg]
	*cps27L*	Deletion	57 bp
	*cps27M*	Missense	C1373T C1374T [Ala458Val]
		Missense	C1375T [Leu459Phe]
vii. Serotype 29: compared to strain YS54 agglutinated with both serotypes 21 and 29 antisera (GenBank accession number KC537387), the insertions of a transposase gene, HG293, HG294, and HG292 on the 3′-side were found in strain 1127863 (Figure 2E).
viii. Serotype 30: compared to the serotype 30 reference strain 92–1400 (GenBank accession number AB737834), insertion of HG9 was found in strains 1611502 and 1839679. Moreover, HG72, transposase gene, and HG73 were replaced by HG17, HG18, and HG19 (Figure 2F).

### Variations of chromosomal loci

In a previous study, the chromosomal loci of *cps* gene clusters of reference serotype 5 and 17 strains were classified into pattern I-a [19]. In the present study, strains 1218846 (serotype 5) and 1224887 (serotype 17) were classified into pattern I-b (Figures 2A and D, respectively).

### MCG typing

The majority of the 79 strains were clustered in the MCG group 6 (44.3%, 35/79 strains), followed by ungroupable (24%, 19/79 strains), and group 7 (20.2%, 16/79 strains). MCG groups 4, 3, 2, and 1 also contained four, three, one, and one strains, respectively (Additional file 1).

### Identifying genotypes of *mrp*, *epf*, and *sly*

Twelve strains were *mrp* positive. Frameshift mutations at 2740 bp from the reported initiator ATG codon were present in the *mrp* gene of strain 1114193, which resulted in premature stop codons. Eleven other strains contained intact full-length *mrp* gene copies and may express MRP. Based on the *mrp* subtypes reported in North America (NA) [11], the sequences of *mrp* were grouped into one of three subtypes, EU (European, *n* = 3), NA1 (*n* = 7), or NA2 (*n* = 1). Only twelve strains contained the *sly* gene and 6 strains were positive for *epf*. There were eight genotypes of *mrp*, *epf*, and *sly*, primarily based on *mrp* variation: most of the strains in this study (*n* = 62) were *mrp* ^−^ *sly* ^−^ *epf* ^−^, followed by *mrp* ^*NA1*^ *sly* ^−^ *epf* ^−^ (*n* = 4), *mrp* ^*NA1*^ *sly* ^+^ *epf* ^−^ (*n* = 4), and *mrp* ^*EU*^ *sly* ^+^ *epf* ^+^ (*n* = 3). It is noteworthy that the latter strains were serotype 2 or 1/2, serotype 15 and serotype 30, as revealed by the 32-plex Luminex assay. In addition, *mrp* ^−^ *sly* ^+^ *epf* ^+^ (*n* = 2), *mrp* ^−^ *sly* ^+^ *epf* ^−^ (*n* = 2), *mrp* ^*NA2*^ *sly* ^+^ *epf* ^−^ (*n* = 1), and *mrp* ^−^ *sly* ^−^ *epf* ^+^ (*n* = 1) genotypes were also found (Additional file 1).

## Discussion

In addition to the traditional 35 serotypes originally described for *S. suis*, 17 NCLs have recently been reported in non-serotypeable *S. suis* strains isolated from healthy animals using high-throughput typing systems and online bioinformatics [22, 23]. However, the genetic characteristics of *cps* loci in potentially virulent non-serotypeable *S. suis* strains recovered from diseased animals are still scarce.

In the present study, the *cps* loci of 79 Canadian non-serotypeable strains isolated from the internal organs of diseased pigs were analyzed. Non-serotypeable strains are frequently isolated from diseased animals in this country [34]. Based on previous gene typing and sequencing results [22, 35], the non-serotypeable phenotype may be attributed to one of three causes: (1) strains belonging to previously described serotypes harboring mutated *cps* loci causing loss of capsule expression or antigenic variation; (2) strains without *cps* locus completely losing their ability to synthesize capsule; or (3) strains with not-previously described NCL referring to novel serotypes.

In this study, 15 non-serotypeable strains could be grouped into reference serotypes by the 32-plex Luminex assay. To elucidate the lack of positive identification by the coagglutination test, we further sequenced and compared their *cps* loci to those of corresponding reference strains. Previous studies showed that replacements and large indels, as well as small-scale mutations of *cps* genes, caused phenotypical changes in agglutination tests [21, 36–38]. We also found similar mutations in the *cps* loci of strains tested.

HG17, HG18, HG19, HG32, HG39, and HG40, which were present in the *cps* loci of the reference strains belonging to serotypes 1, 2, 4, 5, 7, 14, 17, 18, 19, 23 and 1/2, were detected in the *cps* loci of strains in the present study belonging to serotypes 11, 15, and 30. Moreover, chimeric HG18/HG19 and HG32/HG39 genes were found in serotype 11 and 15 strains. It is noteworthy that HG292, HG293, HG294, HG354, and HG355, only present in the *cps* loci of NCLs, were also detected in the *cps* locus of strain 1224889, typed herein as serotype 17. In addition, some genes which were never before assigned to any HG were found to be inserted in the *cps* loci of strains belonging to serotypes 17 and 15. The sequence differences between strains NCL8-2 and 1127863 were mainly caused by the replacement of 8 NCL-specific HGs in the center of NCL8-2 and by 6 HGs in 1127863. The replacement and insertion activities may indicate recombination events or horizontal gene transfer between the *cps* loci of *S. suis* strains, probably leading to antigenic variations that would be beneficial to *S. suis* in the course of infection or through immunity evasion.

Comparing to the *cps* loci of their corresponding reference strains, only small-scale mutations were observed in four strains typed as serotype 2 or 1/2 by the 32-plex Luminex. Previous study revealed that all serotype 2 and all serotype 14 strains had a G nucleotide at position 483 of the *cpsK* gene, while all serotype 1 and all serotype 1/2 strains (including 13 serotype 1/2 strains recovered in Canada) contained either a C or T at that nucleotide position [39]. In present study, all four strains had a G nucleotide at position 483 of the *cpsK* gene. We postulated that they were most probably non-encapsulated serotype 2 strains. A previous study reported that single-nucleotide substitutions and frameshift mutations in two glycosyltransferase genes (*cps2E* and *cps2F*) were the main causes of capsule loss in serotype 2 strains. Moreover, mutations in the genes involved in side-chain formation (*cps2J* and *cps2N*), *wzy* (*cps2I*), and *wzx* (*cps2O*) also appeared to be lethal to serotype 2 strains [36]. It may be hypothesized that the missense mutations and small scale indels found in these genes in strains of the present study also had a deleterious effect on the capsular expression. Indeed, high hydrophobic indexes have been obtained with these strains (unpublished data), which strongly suggest lack of capsule expression [15, 40]. Although non-encapsulated *S. suis* strains had originally been considered to be avirulent, they are frequently isolated from cases of endocarditis; as such, non-encapsulation could be, under certain circumstances, beneficial for *S. suis* in the course of such infections [36, 41]. In some cases, non-encapsulated strains resulting from small point mutations may switch to a capsulated phenotype in vivo [42]. Interestingly, small-scale mutations or clear deletions of *cps* loci were also found in an additional eight strains, which are also probably non-encapsulated. Finally, strains without *cps* locus completely losing their ability to synthesize capsule were also found in this study. It is possible that these strains are not able to reverse the encapsulated phenotype. The biological and pathological significance of these non-encapsulated strains need to be further evaluated. Although never described, it is not impossible that some strains lose the capsular phenotype after in vitro culture.

In this study, 60% of non-serotypeable strains carried one of the recently described NCLs. The most common NCLs were 3, 4, 7, and 17, whereas in a previous study with strains recovered from healthy pigs in China, the most common NCLs were 1, 2, 3, and 7 [22]. Differences may be due to the geographical origin of strains (Canada vs. China) and/or their virulence potential (strains from diseased or clinically healthy animals). Since many strains of NCL3 have been identified in this study, further research on its virulence potential should be performed. In addition, and similarly to a previous study [22], high diversity within the same NCL was observed. The *S. suis* species is composed of phenotypically and genetically diverse strains. Host specificity and ecological environment may contribute to this diversity. The *cps* loci could provide important information regarding the ecology of strains. The differences in dominant NCLs between clinical strains from Canada and field strains from China and the emergence of novel NCLs or subtypes in clinical strains from Canada are expected.

In this study, new NCLs (CNL17–20), distributed in 10 strains, are reported for the first time. These NCLs possess completely different Wzy and transferases from those of the previously reported serotypes and NCLs, which in turn may express unique oligosaccharide structures and antigen identities. It is noteworthy that, taking into consideration all NCLs, more than 70% of non-typeable strains could now be typed. The use of the complete serotyping and NCL typing system would considerably reduce the number of non-typeable strains recovered from diseased animals in Canada.

The presence of some genes, such as *mrp*, *epf*, and *sly*, has been associated with virulence [43, 44]. Three distinct *mrp* genotypes have been reported so far and NA1 was the dominant genotype in *S. suis* strains recovered from diseased pigs in the USA [11]. In the present study, 11 strains possessed an intact *mrp* gene and NA1 genotype whereas three strains harbored the EU genotype. One of latter strains was typed as being a serotype 2 or 1/2 by the 32-plex Luminex but, as mentioned above, it is probably a real serotype 2 as shown by the presence of a G nucleotide at position 483 of the *cpsK* gene. The fact that most *mrp* ^+^, *epf* ^+^, and *sly* ^+^ Eurasian serotype 2 strains belong to the clonal complex 1 [12] also indicated the strain is most probably a non-encapsulated serotype 2 strain with an Eurasian profile [11] that might have been introduced to North America through the importation of animals. In fact, it has been reported that up to 5% of serotype 2 strains recovered in the United States are ST1 and probably originated from Europe [11]. Although the most prevalent virulence gene profile was *mrp* ^−^ *sly* ^−^ *epf* ^−^, 17 strains contained at least one of these three genes. The relevance of these virulence markers in strains of serotypes different from serotype 2 is still controversial.

The most prevalent MCG groups amongst the strains harboring NCLs were the groups 6 and 7, which had been described as being the most ancient groups in the *S. suis* population [30]. This indicates that their *cps* loci have existed for a long time and play important roles in the serotype diversity of *S. suis* population. The most prevalent MCG groups amongst the strains harboring mutated *cps* loci of previously described serotypes and the strains losing their *cps* loci were MCG ungroupable. These strains possibly had a more significant recombination history that prevented them from being reliably assigned; meanwhile these recombination events may facility the mutations and loss of their *cps* loci.

In conclusion, this study provides further insight in understanding the *cps* diversity of *S. suis* and may contribute to future epidemiological studies that will allow characterization of potentially virulent and previously non-serotypeable strains isolated from diseased animals. Use of the 35 serotype-based system complemented with the NCL typing system would significantly reduce the number of untypeable strains recovered from diseased animals in Canada. Further studies with *S. suis* strains isolated in other countries are needed.

## Additional files

**Additional file 1.** **Strains of** ***S. suis*** **used in the present study.** List and identification of strains used in the present study.

**Additional file 2.** **Predicted product and homology group in each NCL.** Predicted product of coding genes and homology group in each characterized NCL.

## Abbreviations

*S. suis*: *Streptococcus suis*
CPS: capsular polysaccharides
NCL: novel *cps* loci
MCG: minimum core genome
*mrp*: muramidase released protein
*sly*: suilysin
*epf*: extracellular protein factor
